# Digital Technologies to Support Better Outcome and Experience of Care in Patients with Heart Failure

**DOI:** 10.1007/s11897-022-00548-z

**Published:** 2022-04-29

**Authors:** K. C. C. McBeath, C. E. Angermann, M. R. Cowie

**Affiliations:** 1 Royal Brompton Hospital (Guy’s & St Thomas’ NHS Foundation Trust), Sydney Street, London, SW3 6NP UK; 2 Comprehensive Heart Failure Centre, University and University Hospital Würzburg, Würzburg, Germany; 3 School of Cardiovascular Medicine, Faculty of Medicine & Lifesciences, King’s College London, London, UK

**Keywords:** Digital technology, Digital health, Heart failure, Shared decision-making, Person-centred care

## Abstract

**Purpose of Review:**

In this article, we review a range of digital technologies for possible application in heart failure patients, with a focus on lessons learned. We also discuss a future model of heart failure management, as digital technologies continue to become part of standard care.

**Recent Findings:**

Digital technologies are increasingly used by healthcare professionals and those living with heart failure to support more personalised and timely shared decision-making, earlier identification of problems, and an improved experience of care. The COVID-19 pandemic has accelerated the acceptability and implementation of a range of digital technologies, including remote monitoring and health tracking, mobile health (wearable technology and smartphone-based applications), and the use of machine learning to augment data interpretation and decision-making. Much has been learned over recent decades on the challenges and opportunities of technology development, including how best to evaluate the impact of digital health interventions on health and healthcare, the human factors involved in implementation and how best to integrate dataflows into the clinical pathway.

**Summary:**

Supporting patients with heart failure as well as healthcare professionals (both with a broad range of health and digital literacy skills) is crucial to success. Access to digital technologies and the internet remains a challenge for some patients. The aim should be to identify the right technology for the right patient at the right time, in a process of co-design and co-implementation with patients.

## Introduction

Digital health encompasses the creation and practical use of computerised devices, methods and systems for health and healthcare [1]. It includes mobile health (mHealth: the use of mobile and wireless technologies to support achieving health objectives [2]), health information technology (IT), wearable devices and remote patient management (RPM). Digital health is an integral part of the transformation of healthcare systems towards a more patient-centric preventive model, where resources are focused toward health maintenance rather than only on crisis management.

The vision of a person-centred healthcare system is key to policy makers’ plans for a more modern, flexible and sustainable healthcare system [3, 4], where people living with (or at risk of) cardiovascular disease use a range of digital technologies to ensure optimal experience and outcome of health (and healthcare) decision-making, only interacting with the appropriate healthcare professionals when (and if) this is useful.

The implementation of digital systems in healthcare such as electronic medical records and e-prescribing has accelerated in recent years [5] but technologies to support health maintenance and healthcare decision-making (such as remote monitoring) have seen slower uptake. The COVID-19 pandemic has triggered a rapid wave of adoption and greater acceptance of digital technologies by patients, healthcare professionals (HCP) and systems — a process often referred to as a “tech-celleration” — as change that was expected to take years occurred within a few weeks in response to the need for restricted face-to-face interaction [6•].

**Fig. 1:**
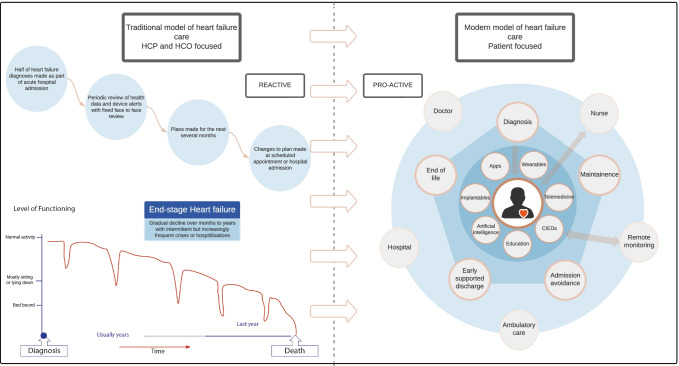
The contrast between the traditional model of heart failure care (left), with a modern digitally-supported patient-centric model of heart failure care (right). The traditional model uses fixed periodic review with a healthcare organisation (HCO) focus on reactive management of patient crises. The modern model of heart failure care uses digital technology to support patients at various stages of their heart failure illness, allowing dynamic management of issues as they arise. Healthcare organisation focus is on pro-active health maintenance. HCP, healthcare professional; HCO, healthcare organisation; CIEDs, cardiac implantable electronic devices; Apps, smartphone applications

This article reviews the lessons learned in the creation, assessment and implementation of digital health technologies for heart failure (HF) management, focusing on remote monitoring and devices to support and improve patient and HCP decision-making (remote patient management, RPM) compared with traditional HF care strategies (Fig. 1). We also share our vision of the future, where the right tool is used at the right time and in the right place to enable optimal outcome and experience of care.

## Lessons Learned from Remote Monitoring Using Stand-Alone Technologies

**Fig. 2:**
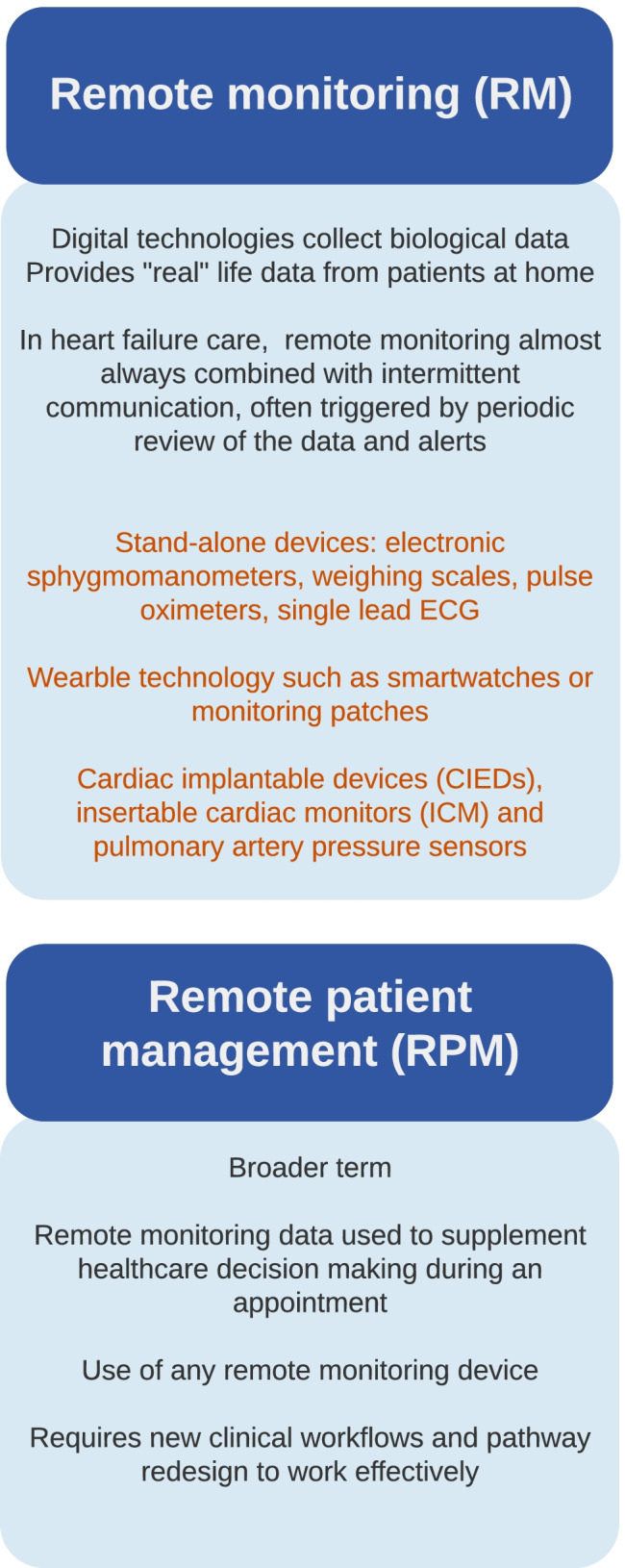
What are remote monitoring and remote patient management?

The early remote monitoring studies for patients with HF tended to be small, often single centred and of short duration. Patients were typically recruited at (or shortly after) a heart failure hospitalisation (HFH), and the technologies used were simple and stand-alone. Some of the key studies are listed in more detail in (Table 1). Such studies are likely to be subject to publication bias (positive studies more likely to be published than neutral or negative studies) and may not have been representative of more routine practice due to the enthusiasm of the single centres (Fig. 2).

**Table 1:** Trials with standalone devices and those with rehabilitation

	*Year of publication* *Type of study*	*Location of study* *No. of centres*	*No. of patients* *Intervention group* *Control group*	*Length of follow-up* *Mean* ± *SD OR Median (IQR)*	*Age* *Mean* ± *SD OR Median (IQR)* *Sex % female*	*Inclusion criteria*	*Main exclusion criteria*	*Equipment used*	*Intervention*	*Primary (1º) endpoint* *Main secondary (2º) endpoints*	*Effect size*
*Tens-HMS *[7]	2005 RCT	Europe UK 16	426 163 RM 170 STS 85 UC	484 (317- 622) days	67 years 22% female	LVEF < 40%, LVEDd > 30 mm/m HFH in last 6 weeks Furosemide ≥ 40 mg/day	Unable to comply with RM awaiting revasc or CRT or HTx	Home hub with scales, BP, single lead ECG	Twice daily RM data sent centrally OR STS	1º: Days lost because of death or hospitalisation with RM vs STS at 240 days 2º: All-cause mortality, optimisation of medication with RM vs STS	1º: 4898 days lost in RM group vs 6389 STS, − 8 days difference between means (95% *CI* − 25 to 10) (*p* = not significant) 2º: No significant differences observed RM + STS significantly reduced rate of mortality and fewer days lost vs UC
*HOME-HF *[8]	2009 RCT	UK 3	182 91 RM 91 UC	6 months	72 ± 12 years 34% female	Any LVEF NYHA II-IV at discharge from hospital	Cognitive impairment	HomMed device with scales, BP, Sp0_2_	Daily RM data including answers to 4 automated questions Reviewed 5 × weekly	1º: Number of days alive and out of hospital 2º: Number and duration of HFH	1º: Not statistically significant. Median of 178 (*IQR* 90–180) days in RM group vs 180 days in UC group (*IQR* 165–180) *p* = − 0.30 2º: Not statistically significant. 17 patients HFH in RM group vs 10 STS group. 17-day HFH duration in RM vs 9 STS
*TELE-HF *[9]	2010 RCT	US 33 sites	1653 826 RM 827 UC	6 months	61 (53–73) years 42% female	Any LVEF Any NYHA HFH in last 30 days	NH resident < 6-month survival Severe cognitive impairment	Scales	Daily RM data and phone calls with responses to automated questions	1º: Composite of all-cause readmission or all-cause mortality 2º: Hospitalisation, mortality, number of days in hospital, number hospitalisation	1º: Not statistically significant. 432 patients RM vs 426 patients UC. *HR* 1.04 (95% *CI*, 0.91 to 1.19) 2º: Not statistically significant. Readmission HR in RM group 1.06 (95% *CI*, 0.93 to 1.22). Mortality HR for RM 0.97 (95% *CI*, 0.73 to 1.30)
*TIM-HF *[10]	2011 RCT	Germany 165 sites	710 354 RM 356 UC	26 (12–28) months	67 ± 11 years 19% female	LVEF ≤ 35% NYHA II-III HFH in last 24 months OR LVEF ≤ 25%	Life expectancy < 1 year (excluding HF), awaiting cardiac intervention	Wireless digital assistant with bluetooth scales, BP, 3-lead ECG	Daily RM data to central location + STS + 24/7 physician led call centre	1º: All-cause mortality 2º: Composite of cardiovascular (CV) death + HFH	1º: Not statistically significant. Rate per 100 person-years of 8.4% in RM vs 8.7% in UC (*HR* 0.97; 95% *CI* 0.67–1.41; *p* = 0.87) 2º: Not statistically significant. Rate per 100 person-years of 14.7% in RM vs 16.5% in UC (*HR* 0.89; 95% *CI*, 0.67 to 1.19; *p* = 0.44)
*BEAT-HF *[11]	2016 RCT	US 6	1437 715 RM 722 UC	6 months	73 (63–83) years 46% female	Current HFH or receiving active treatment for WHF > 50 years old	Severe cognitive or physical condition Awaiting cardiac intervention ESRF	Wireless transmission assistant with bluetooth scales, BP, HR, simple question and answer device	Education Telephone coaching sessions Daily RM data to nurse led call centre	1º: 180 day all-cause readmission 2º: 30-day all-cause readmission, 30-day mortality, and 180-day mortality	1º: Not statistically significant. Readmissions: 363 (50.8%) in RM group vs 355 (49.2%) in UC (*HR*, 1.03; 95% *CI*, 0.88-0.20; *P* = 0.74) 2º: No significant differences Significant difference in quality of life between RM vs UC
*TIM-HF2 *[12••]	2018 RCT	Germany 113 sites	1571 796 RM 775 UC	1 year	70 ± 11 years 31% female	LVEF ≤ 45% or higher if on oral diuretics NYHA II-III HFH in last 12 months	Major depression, ESRF, hospitalisation in last 7 days, intervention in last 28 days	Wireless digital tablet + 3-lead ECG, BP, scales, SpO_2_	Daily RM data to central location + STS + 24/7 physician led call centre	1º: Percentage of days lost due to unplanned CV hospitalisation or all-cause mortality 2º: All-cause mortality and CV mortality, change in MLHFQ, change in NT-proBNP	1º: RM significantly reduced percentage of days lost 4.88% (95% *CI*, 4.55–5.23) in RM group vs 6.64% (6.19–7.13) in UC. (*HR* 0·80, 95% *CI*, 0.65–1.00; *p* = 0.0460*) 2º: Significantly reduced all-cause death rate 7·86 (95% *CI* 6.14–10.10) per 100 person-years in RM group vs 11.34 (9.21–13.95) in UC (*HR* 0.70, 95% *CI* 0.50–0.96; *p* = 0.0280*)
*Inglis *et al. [13]	2010 Meta-analysis of 30 RCT − 11 RM − 16 STS	Intl 14 US 10 EU 4 other	8323 2710 RM 5613 STS	3–18 months	Mean 45–78 years 36% (1–65) female	Peer reviewed RCTs comparing STS or RM to UC	Home visits or more than usual (4–6 week) follow-up	Various	Meta‐analysis using fixed effects models	1º: All‐cause mortality 2º: All‐cause and HF hospitalisation, length of stay, quality of life, acceptability and cost	1º: RM significantly reduced all‐cause mortality (*RR* 0.66, 95% *CI*, 0.54 to 0.81, *P* < 0.0001*) vs UC 1º: STS showed a non‐significant positive effect (*RR* 0.88, 95% *CI*, 0.76 to 1.01, *P* = 0.08) vs UC 2º: Both RM (*RR* 0.79, 95% *CI* 0.67 to 0.94, *P* = 0.008*) and STS (*RR* 0.77, 95% *CI* 0.68 to 0.87, *P* < 0.0001*) significantly reduced HF‐related hospitalisations
*Zhu *et al. [14••]	2020 Meta-analysis of 29 RCT - 19 RM - 9 STS - 1 both	Intl	10,000	11 (1–36) months	66 years 33% female	LVEF < 45% NYHA I–IV	nil	Various	Meta-analysis	1º: All-cause mortality and all-cause hospitalisation 2º: HFH	1º: Significant reduction mortality (*OR* 0.75, 95% *CI* 0.62–0.90, *P* = 0.003*) and hospitalisation (*OR* 0.82, 95% *CI* 0.73–0.91, *P* = 0.0004*) in RM group vs UC 2º: Significant reduction HFH in RM group vs UC (*OR* 0.83, 95% *CI* 0.72–0.95, *P* = 0.007*)
*HF-ACTION *[15]	2009 RCT	US Canada France 82	2331 759 exercise 796 UC	30 (12–48) months	59 (51–68) years 28% female	LVEF ≤ 35% NYHA II-IV Despite OMT 6 months	Major comorbidity or limitation Recent or planned major CV events or procedure regular exercise	Home cycle or treadmill (ICON) + heart rate monitor (Polar USA)	12 -week Structured & supervised group-based home exercise 3 × weekly 36 sessions	1º: composite of all-cause mortality or all-cause hospitalisation 2º: included all-cause mortality, composite of CV mortality or HFH	1º: Not statistically significant. 759 patients in the exercise training group (65%) vs 796 patients in UC group (68%) experienced a primary clinical event (*HR* 0.93 [95% *CI*, 0.84–1.02]; *P* = 0.13). Absolute reduction in the event rate at 3 years was 4%
*REACH-HF *[16]	2019 RCT	UK 4	216 107 Reach 109 UC	12 months	70 ± 11 years 22% female	LVEF < 45%	Rehab in last 12 months	nil	12-week REACH-HF telephone and face-to-face Exercise ≥ 3 × weekly Progress tracker Family resource and HCP support	1º: MLHFQ 2º: death, hospitalisation, EQ-5D-5L, HADS	1º: Significantly reduced MLHFQ − 5.7 points (95% *CI* − 10.6 to − 0.7, *p* = 0.025*) in favour of the REACH-HF group 2º: Significant improvement in maintenance section of self-care 63.8 ± 17 vs 55 ± 16 — difference of 8.0 (95% *CI* 3.6 to 12.4; *p* < 0.001*)
*TELEREH-HF *[17•]	2020 RCT	Poland 5	850 425 Rehab 425 UC	14–26 months	63 ± 11 years 11% female	LVEF ≤ 40% NYHA I-III HFH in last 6 months	MI < 40 days with LVEF < 35% PCI in last 2 weeks CABG in last 3 months	Scales, BP, 3 lead ECG + RM from CIED if available	9-week Hybrid comprehensive telerehabilitation (HCTR) Week 1 hospital Week 2–9 home 5 × weekly	1º: Percentage of days alive and out of hospital during 14–26-month follow-up 2º: all-cause mortality and CV mortality. All-cause, CV and HFH	1º: Not statistically significant. Probability that HCTR extends days alive and out of hospital 0.49 (95% *CI*, 0.46–0.53; *P* = 0.74) 2º: Not statistically significant. Mortality rate 12.5% with HCTR vs 12.4% with UC (*HR* 1.03, 95% *CI* 0.70–1.51) 3º Large significant improvement in 6MWT, VO2 max, NYHA class at 9 weeks
*REHAB-HF *[18]	2021 RCT	US 3	349 175 Rehab 174 UC	6 months	73 ± 8 years 52% female	Any LVEF Any NYHA Current HFH > 60 years Can walk > 4 m	Acute MI Discharge to NH Regular exercise	nil	12-week home programme for frail people with HF 3 × weekly 36 sessions	1º: score on the Short Physical Performance Battery (SPPB) at 3 months 2º: 6-month rate of all-cause rehospitalization	1º: Significant improvement in SPPB 8.3 ± 0.2 vs 6.9 ± 0.2 in rehab vs UC (mean between-group difference, 1.5; 95% *CI*, 0.9 to 2.0; *P* < 0.001*) 2º: Not statistically significant. Rates of rehospitalization 1.18 in rehab group vs 1.28 in UC (rate ratio, 0.93; 95% *CI*, 0.66 to 1.19)

*No*, number; *SD*, standard deviation; *IQR*, interquartile range; *RCT*, randomised controlled trial; *RM*, remote monitoring; *STS*, structured telephone support; *UC*, usual care; *LVEF*, left ventricular ejection fraction; *LVEDd*, left ventricular end-diastolic dimension; *HFH*, heart failure hospitalisation; *CRT*, cardiac resynchronisation therapy; *HTx*, heart transplant; *BP*, blood pressure; *CI*, confidence interval; *HR*, hazard ratio; *NYHA*, New York Heart Association classification of heart failure; *Sp02*, oxygen saturation; *NH*, nursing home; *WHF*, worsening heart failure; *ESRF*, end stage renal failure; *MLHFQ*, Minnesota Living with Heart Failure Questionnaire; *Intl*, International; *HF*, heart failure; *CV*, cardiovascular; *EQ-5D-5L*, five-dimension European Quality of Life scale; *HADS*, hospital anxiety and depression scale; *OMT*, optimal medical therapy; *MI*, myocardial infarction; *PCI*, percutaneous coronary intervention; *CABG*, coronary artery bypass grafting

Meta-analysis of these early small randomised studies suggested a powerful impact on both mortality and hospitalisation compared to usual care [13], with a 34% relative risk reduction in mortality (*CI* 19–46%, *p* < 0.0001) and 21% relative risk reduction in heart failure hospitalisation (HFH) (*CI* 6–33%, *p* = 0.008). How this effect was mediated is unclear but is likely to include improved guideline compliance by HCPs, improved medication and lifestyle compliance by patients, and earlier detection of decompensation with intervention to restabilise the HF syndrome and reduce the need for hospitalisation.

Except for the encouraging results of two early larger trials [7, 19], the next generation of multicentre studies (consequently adequately powered, arguably more likely to be representative of usual practice, and less subject to publication bias) often failed to show a significant difference in “hard” endpoints such as mortality or HFH over 6–20 months of follow-up. This was despite a variety of patient phenotypes and risk, and a wide range of interventions including devices with embedded reminders and assessment of patient-reported symptoms, structured telephone support with nurse specialists and monitoring of weight, blood pressure, heart rate and ECG [8, 9, 20].

TIM-HF2 (Telemedical Interventional Management in patients with Heart Failure) provided the first positive large randomised controlled trial (RCT) in RPM using stand-alone equipment in 710 patients with HF, New York Heart Association (NYHA) class II or III, and a HFH within the preceding 12 months.

TIM-HF2 reported a borderline statistically significant 20% reduction (*p* = 0.046) in the primary endpoint of percentage of days lost to unplanned admission or death during 12-month follow-up from 6.64 to 4.88%, with no statistically significant reduction in the secondary endpoint of cardiovascular (CV) mortality (*p* = 0.056) [12••]. However, it required an intensive and well-resourced approach, with daily review of the monitoring data, ability to stream ECGs and a centralised 24/7 telemonitoring centre in Berlin with close connection with the emergency services, local general practitioners and the patient and their family. Assessment of the likely cost-effectiveness of this approach, suggested a small cost saving per patient year (€1760) in the German healthcare system [21].

The neutral results from several other large RCTs, and the selected nature of the population within TIM-HF2, led to the most recent European Society of Cardiology (ESC) guidelines to be lukewarm about its recommendation for routine telemonitoring of patient with HF with stand-alone equipment (class 2 level B — “may be considered”). [22]. Similarly, weak recommendations have been issued by the American, Canadian and Australian HF societies [23–25].

Rehabilitation and training programmes in HF have used a combination of supervised and unsupervised sessions in hospital and at home. TELEREH-HF (The Telerehabilation in Heart failure patients) RCT randomised 850 patients up to 6 months after a HFH with NYHA I-III symptoms and reduced ejection fraction [17•]. A total of 425 patients received a 9-week hybrid comprehensive telerehabilitation (HCTR) programme encompassing RPM and individualised tele-rehabilitation. This hybrid approach did not extend the percentage of days alive and out of hospital at extended follow-up: 91.9 (± 19.3) HCTR group vs 92.8 days in the usual care group (± 18.3)(*p* = 0.74). However, several physical rehabilitation studies have replicated the other findings of TELEREH-HF, with significant but short-term improvement in symptoms, physical performance, functional capacity and depressive symptoms [16, 18, 26, 27] without translating into reductions in mortality or HFH risk [15, 17•]. It is likely that cardiac rehabilitation and physical fitness need to be maintained in order to gain long-term benefits [28] and may be dependent on improving patient’s capacity for self-management [17•]. Ongoing support by digital technology and remote patient management may facilitate these objectives.

## Lessons Learned from Remote Monitoring and Remote Patient Management Using Implantable Devices

### Cardiac Implantable Electronic Devices (CIEDs)

CIEDs record multiple physiological variables — such as heart rate, heart rate variability, episodes of arrhythmia including atrial fibrillation or fast ventricular rhythms, transthoracic impedance, patient activity, and in some systems sleep apnoea and the intensity of heart sounds. Many of these variables are associated with the risk of HF decompensation [29, 30].

Initial studies (detailed in Table 2) likely placed too much weight on one variable leading to too many false positives. In the DOT-HF (Diagnostic Outcome Trial in Heart Failure) randomised study, an audible alarm was triggered within the device if a threshold of a decreasing trend in transthoracic impedance (reflection of increasing lung water and left ventricular filling pressure) was crossed. This triggered a 79% increase in HF hospitalisation, and the study was stopped early due to this [31]. The human factors triggered by an audible alarm sounding within the device were likely part of the problem: it is difficult for the patient, the family, and the emergency room physicians to ignore an alarm. Many of the admissions were short and with low mortality — suggesting that HF had not truly decompensated, and the admission was often unnecessary.

**Table 2:** Trials with cardiac implantable electronic devices (CIEDs)

	*Year of publication* *Type of study*	*Location of study* *No. of centres*	*No. of patients* *Intervention group* *Control group*	*Length of follow*-*up* *Mean* ± *SD* *OR Median (IQR)*	*Age* *Mean* ± *SD* *OR Median* *(IQR)* *Sex % female*	*Inclusion criteria*	*Main exclusion criteria*	*Equipment used*	*Intervention*	*Primary (1º) endpoint* *Main secondary (2º) endpoints*	*Effect size*
*DOT-HF *[31]	2011 RCT	Intl 72	335 168 RM 167 UC	15 ± 5 months	64 ± 10 years 14% female	LVEF ≤ 35% NYHA II-IV HFH in last 12 months	Scheduled or recent cardiac surgery or HTx MI < 40 days, life expectancy < 1 year	CIED Medtronic	Audible alert for patients triggered clinical review and review of RM data by physician	1º: Composite of all-cause mortality or HFH 2º: All-cause mortality, HFH	1º: Increase in CVE in RM group. 48 vs 33 events in UC (*HR* = 1.52; 95% *CI*, 0.97–2.37; *p* = 0.063*) 2º: Not statistically significant. Mortality 19 in RM group vs 15 in UC (*HR* = 1.24; 95% *CI*, 0.63–2.44; *p* = 0.54) 2º: HFH significantly increased 60 in RM group (41 patients) vs 36 in UC (24 patients). (*HR* = 1.79; 95% *CI*, 1.08–2.95; *p* = 0.022*)
*SENSE-HF *[32]	2011 Cohort study	Intl 41	501 Phase I 371 Phase II/III	1.3 ± 0.7 years	65 ± 10 years 15% female	Any LVEF Any NYHA HFH in last 12 months CIED implant in last 34 days	Not supplied	CIED Medtronic	Phase I blinded to optivol Phase II/III: Optivol guided patient Mx with audible patient alert	1º: Phase I: HFH within 30 days of Optivol threshold crossing 1º: Phase II: PPV of first Optivol alert for detection of WHF	1º: Phase I: 12 of 58 HFH preceded by Optivol threshold crossing — sensitivity 20.7% and PPV 4.7% of Optivol index for predicting HFH 1º: Phase II: 210 of 233 optivol alerts were followed by evaluation of HF status. 80 of these had WHF — PPV 37.9%
*Heart Failure Risk Score (HFRS) *[33]	2013 Develop AND validate CIED risk algorithm	Intl Data from 6 trials	921 develop set 1310 validate set	11 ± 6 months	68 ± 11 years 28% female	> 90 days of CIED data	Permanent AF or severe comorbidity	CIED Medtronic	HFRS alert algorithm	1º: Survival free from HF events in the 30 days after a HFRS alert	High HFRS group were 10 times (adjusted HR: 10.0; 95% *CI*: 6.4–15.7, *P* < 0.001*) more likely to have an HFH (event rate 6.8%) in the next 30 days vs low HFRS group (event rate of 0.6%)
*IN-TIME *[29]	2014 RCT	Europe (Israel) (Australia) 36	664 333 RM 331 UC	12 months	66 ± 10 years 19% female	LVEF ≤ 35% NYHA II-III OMT Recent CIED	Permanent AF or severe comorbidity	ICD or CRT-D Biotronik	Daily RM data, reviewed centrally. Response at clinician’s discretion	1º: composite all-cause mortality + HFH + change in NYHA + change in patient global self-assessment score	1º: Significant reduction. 63 patients (18·9%) in RM vs 90 (27·2%) in UC group (*p* = 0·013*) had worsened composite score (odds ratio 0·63, 95% *CI* 0·43–0·90)
*OptiLink *[34]	2016 RCT	Germany 65	1002 505 RM 497 UC	23 ± 18 months	66 ± 10 years 20% female	LVEF ≤ 35% NYHA II-III HFH in last 12 months OR diuretics in last 30 days OR raised natriuretic peptides	ESRF, severe COPD, planned HTx	CIED Medtronic	Automatic fluid index alerts + pre-specified Mx algorithm	1º: Composite all-cause mortality + CV hospitalisation 2º: All-cause mortality, CV mortality	1º: Not statistically significant. 227 patients in RM group vs 239 in UC, event free survival 52.7 vs 47.8% (*HR*, 0.87; 95% *CI*, 0.72–1.04; *P* = 0.13) 2º: Not statistically significant. All-cause mortality 11.0% RM vs 15.7% UC (*HR*, 0.89; 95% *CI*, 0.62–1.28; *P* = 0.52)
*REM-HF *[35]	2017 RCT	UK 9	1650 826 RM 824 UC	2.8 (0–4.3) years	70 ± 10 years 14% female	Any LVEF NYHA II-IV OMT for 6 weeks CIED implant in last 6 months	Device intervention in last 30 days, MI or cardiac procedure in last 3 months	CIED Medtronic, Boston scientific & St Jude	Weekly RM data with standardised clinical Mx handbook	1º: Composite of mortality or CV hospitalisation 2º: all-cause mortality, CV mortality, HFH	1º: Not statistically significant. 349 patients (42.4%) in RM group vs 347 (40·8%) in UC group (*HR* 1.01; 95% CI 0.87 to 1.18; *P* = 0.87) 2º: No significant difference
*MORE-CARE *[36]	2016 RCT	Europe Israel 61	918 426 RM 455 UC	24 (15–25) months	66 ± 10 years 24% female	CRT-D implant in last 8 weeks	Permanent AF, Life expectancy < 1 year	CRT-D Medtronic	Automatic RM alerts + UC including 4 monthly face-to-face follow-up	1º: Composite of mortality + CV and device-related hospitalisation (> 48 h) 2º: utilisation of resources for CV care	1º: Not statistically significant. 130 events (29.7%) in RM arm vs 123 (28.7%) in UC arm: Kaplan–Meier 2-year risk estimates 34.3% (95% *CI* 29.7–39.4) vs 32.7% (95% *CI* 28.2–37.8), respectively (*P* = 0.89) 2º: Significantly reduced 38% reduction in costs incidence rate ratio (IRR) 0.62, 95% *CI* 0.58–0.66, *P* < 0.001*)
*MultiSENSE *[37]	2017 Develop and validate CIED risk algorithm	Intl 93	974 531 develop set 443 test set	12 months	67 ± 10 years 28% female	Any LVEF NYHA II-IV CRT-D implant	Nil significant	CRT-D Boston Scientific	Heartlogic multisense index and alert algorithm	1º: Validate the algorithm for sensitivity of detecting HF events 1º: Rate of unexplained alerts per patient-year	1º: Algorithm sensitivity of 70% with a median alert window of 34 days before HF event 1º: Unexplained alert rate of 1.47 per patient-year
*MultiSENSE *post hoc* analysis *[38]	2018 Post hoc analysis	Intl 93	974 531 develop set 443 test set	12 months	67 ± 10 years 28% female	Any LVEF NYHA II-IV CRT-D implant	Nil significant	CRT-D Boston Scientific	Heartlogic multisense index and alert algorithm		IN HeartLogic alert state event rate of 0.8/patient-year vs OUT OF-alert 0.08/patient -years (Event rate ratio 7.05 [95% *CI*. 4.69–10.61; *p* < 0.0001*]) IN-alert + NT-proBNP > 1000 had a 50 × increased risk of HF events (1.00 events/pt-yr) relative to the low-risk group (0.02 events/pt-yr)
*Triage-HF *[39]	2018 Cohort study	Canada 3	100	8 months	67 ± 11 years 22% female	Any LVEF Any NYHA CRT-D or ICD implant	System modification at any time during study	CIED Medtronic	Telephone triage within 24 h of High HFRS (Medium HFRS at clinicians’ discretion)	1º: Correlate high HFRS with signs, symptoms, and behaviours associated with WHF 2º: Evaluate medium HFRS who were contacted by telephone	1º: Signs/symptoms WHF and non‐compliance identified in 83–85% of patients with high HFRS (*n* = 24) 2º: In medium HFRS, 8% had WHF or non‐compliance (29 of 368 patients). When just the 31 patients who were contacted were considered, it rose to 94%
*Triage-HF plus *[40]	2020 Cohort study	UK 1	231 118 High HFRS 113 medium/low HFRS	27 months	70 ± 14 years 45% female	Any LVEF Any NYHA CIED implant	Patients with a high‐risk HFRS who we were unable to be contacted by telephone	CIED Medtronic	5 triage screening questions in response to high HFRS Positive screening questions = “triage positive”	1º: Diagnostic accuracy of the HFRS to identify WHF — comparing high HFRS with clinical diagnosis made by HCP	1º: 90 (71%) of 127 contactable patients were “Triage positive”. 71 diagnosed with WHF (alone or alongside an acute medical problem) requiring medical intervention Sensitivity and specificity of a high HFRS to identify WHF 98.6% (92.5–100.0%) and 63.4% (55.2–71.0%), respectively. Overall accuracy 74.7% (68.5–80.2%)
*SELENE HF *[41]	2021 Develop and validate CIED risk algorithm	Italy Spain 34	918 457 develop set 461 validate set	23 (14–36) months	69 (61–76) years 19% female	LVEF ≤ 35% NYHA II-III CRT-D or ICD implant	AF WHF	CIED Biotronik	Daily RM data combined with baseline risk stratifier: SHFM	1º: First post-implant HFH 2º: HF event rate composite of HFH, IV diuretics, HF mortality	1º: 65.5% of HF events could be predicted (*CI* 45.7–82.1%). Median alert time 42 days, false alert rate 0.69 alerts per patient-year, and unexplained alert rate 0.63 per patient-year

*No*, number; *SD*, standard deviation; *IQR*, interquartile range; *RCT*, randomised controlled trial; *Intl*, International; *RM*, remote monitoring; *UC*, usual care; *LVEF*, left ventricular ejection fraction; *NYHA*, New York Heart Association classification of heart failure; *HFH*, heart failure hospitalisation; *HTx*, heart transplant; *MI*, myocardial infarction; *CIED*, cardiac implantable electronic device; *HR*, hazard ratio; *CI*, confidence interval; *WHF*, worsening heart failure; *ESRF*, end stage renal failure; *CRT*, cardiac resynchronisation therapy; *MLHFQ*, Minnesota Living with Heart Failure Questionnaire; *HF*, heart failure; *CV*, cardiovascular; *EQ-5D-5L*, five-dimension European Quality of Life scale; *HADS*, hospital anxiety and depression scale; *OMT*, optimal medical therapy; *PCI*, percutaneous coronary intervention; *CABG*, coronary artery bypass grafting; *AF*, atrial fibrillation; *COPD*, chronic obstructive pulmonary disease; *Mx*, management; *HCP*, healthcare professional; *SHFM*, Seattle heart failure model

In the Optilink-HF Study (Optimization of Heart Failure Management using OptiVol™ Fluid Status Monitoring and CareLink™) in just over 1000 patients followed up for an average of 23 months, few actions were taken in response to “alerts” on changes in transthoracic impedance sent by SMS to the responsible physicians, and when action was taken, it was often delayed [42]. There is little point in remotely monitoring a patient if the data collected are not rapidly integrated into the decision-making processes.

With the disappointing results from single parameter monitoring, investigators moved to a more broadly based approach, with “multiparametric” monitoring, often with an algorithm-based approach to stratifying patients into three risk categories (high, medium or low) [33, 38]. In patients stratified into the highest-risk group, the absolute risk of HF decompensation within the next month is low (7% in one analysis [42]), implying that many patients will restabilise even if algorithmic interpretation of the parameters is reliable.

The IN-TIME (Implant-based Multiparameter Telemonitoring of Patients with Heart Failure) study [29] evaluated automatic daily data transmission of multiparametric device data to a single call centre vs. those receiving conventional follow-up through 12 months after ICD or CRT-D implantation in 716 patients enrolled at 36 centres in Australia, Europe and Israel [43]. The odds of the primary endpoint of the composite “Packer” clinical score for HF, (comprising all-cause death, overnight HFH, change in NYHA class, and change in patient global self-assessment) worsening in the intervention group was 0.63 (95% *CI* 0.43–0.90) compared with the control group. A reduction in a secondary endpoint of all-cause mortality was also reported. The authors interpreted their findings as most likely due to the centralized daily review of all monitored parameters, combined with the protocolised and timely actions that were taken in response to the data [29]. A pooled analysis of remote monitoring of CIEDs using one manufacturer’s technology (including IN-TIME) reported that active remote management was associated with a 36% reduction in HFH (*p* = 0.007) and a borderline significant reduction in all-cause (but not CV) mortality [44].

In the larger and longer duration REM-HF (REmote Management of Heart Failure using implantable electronic devices) study at nine large UK hospitals, all the remotely collected data from a CIED was reviewed weekly by a team experienced in HF and remote monitoring [35]. They had the time to focus on the remote monitoring processes and worked to a standardised protocol. Despite multiple actions being taken by the monitoring teams in response to the data review of the 1650 patients followed up for a mean of 2.8 years, there was no significant change in either HFH or CV mortality. This study illustrates that without prespecified standard operating procedures, it is not straightforward to translate complex multiparametric monitoring information into “actionable” care strategies to improve clinical outcomes.

More formal approaches to multiparametric monitoring include the Triage-HF studies [39, 40]. In the British Triage-HF plus, a high “Heart Failure Risk Score” (HFRS™) triggered a clinical phone triage system operated by the local HF team. This approach provided high sensitivity but low specificity for worsening HF. The investigators concluded that it was safe to continue to monitor remotely patients with medium or low risk scores, but those with a high-risk score required telephone triage, with 71% being positive for symptoms of worsening HF or an alternative medical problem. Most recently, the HeartLogic™ algorithm has been tested in an FDA-approved study. MANAGE-HF (Multiple cArdiac seNsors for mAnaGEment of Heart Failure) — phase I [45•] uses a scoring system developed and validated from large multicentre datasets, but with additional support for centres to continually review and act on the data and to increase their efforts to persuade patients to act on the advice given. It is clear that if a patient is not willing to change their medication or lifestyle advice compliance in response to remotely collected data, the link between more data and better outcome will be lost.

The role of insertable cardiac monitors (ICMs) in HF care has been limited currently to the diagnosis of arrhythmia such as sub-clinical atrial fibrillation [46]. ALLEVIATE-HF (Algorithm Using LINQ Sensors for Evaluation And Treatment of Heart Failure: NCT04452149), due to report in 2024, will randomise up to 700 patients to an ICM-based risk stratification algorithm (plus a medicines management plan) or usual management. The endpoint is a hierarchical composite of cardiovascular death, HF events, change in Kansas City Cardiomyopathy Questionnaire and change in 6-min walk test distance.

### Remote haemodynamic monitoring

Remote haemodynamic monitoring has been examined with a range of technologies in the past two decades (described in detail in Table 3). Most data are related to the implantable pulmonary artery pressure (PAP) monitoring system CardioMEMS™ HF. Patients receive a home Patient Electronics Unit for daily upload of resting, supine PAP information from the sensor to a secure website (Merlin.net™). PAP increases represent an early sign of imminent cardiac decompensation. Uploaded PAP information should be reviewed at least weekly by trained HCPs. Additional PAP reviews are triggered by email notifications of PAP excursions outside the user-defined thresholds automatically issued by the Merlin.net system. The sensor is easily implanted at right heart catheterisation, carries a low risk of technical failure in routine care [47•, 48] and facilitates targeting of a specific PAP range, with adjustment of HF therapies including diuretics to maintain patients within that range, where possible. Most adjustments occur in the first few months of monitoring, and thereafter, trends in the pressures are used to detect signs of decompensation or over-treatment.

**Table 3:** Trials with invasive monitoring

	*Year of publication* *Type of study*	*Location of study* *No. of centres*	*No. of patients* *Intervention group* *Control group*	*Length of follow*-*up* *Mean* ± *SD* *OR Median (IQR)*	*Age* *Mean* ± *SD* *OR Median* *(IQR)* *Sex % female*	*Inclusion criteria*	*Main exclusion criteria*	*Equipment used*	*Intervention*	*Primary (1º) endpoint* *Main secondary (2º) endpoints*	*Effect size*
*COMPASS-HF *[49]	2008 RCT	US 27	277 134 RM 140 UC	6 months	58 ± 14 years 34% female	LVEF < 50% NYHA III-IV HFH in last 6 months	Severe COPD, PAH, CVE in last 3 months, ASD/VSD, tricuspid or pulmonary stenosis, mechanical heart valves	Right ventricle continuous haemodynamic monitor Chronical Medtronic	Weekly haemodynamic data used to guide patient Mx	1º: HF events — HFH and urgent HF hospital visits 1º: Safety endpoint: freedom from system-related or pressure sensor complications	1º: HF event rate per 6 patient months 0.67 in RM and 0.85 in UC. Non-significant reduction of 21% in rate of HF events (*p* = 0.33) 1º: Complication-free rate of 91.5% (one-sided 95% *CI* of 88.7%). 83% (*n* = 20) of events successful resolved
*CHAMPION *[50]	2011 RCT	US 64	550 270 RM 280 UC	6 months	61 ± 13 years 27% female	Any LVEF NYHA III	Recurrent VTE CIED in last 3 months eGFR < 25 ml/min	Pulmonary artery pressure sensor CardioMEMS, Abbott	Daily PAP data used to guide patient Mx	1º: Rate of HFH at 6 months 1º: Safety endpoint device or system related complication	1º: 84 HFH in RM group vs 120 in UC group. Event rate 0.32 in RM vs 0.44 in UC, *HR* 0.72, 28% significant reduction in HFH (*HR* 0.72, 95% *CI* 0.6–0.85, *p* = 0.0002*) 1º: 98.6% (95% *CI* 97·3–99·4) freedom from complication
*CHAMPION fu *[51]	2016 RCT	US 64	347 177 RM 170 UC	18 months	61 ± 13 years 27% female	Any LVEF NYHA III	Recurrent VTE CIED in last 3 months eGFR < 25 ml/min	CardioMEMS Abbott	Daily PAP data used to guide Mx	1º: Rate of HFH at 18 months	1º: Significantly reduced HFH in RM group (*HR* 0·67 [95% *CI* 0·55–0·80]; *p* < 0·0001*) vs UC
*GUIDE-HF *[52]	2021 RCT Single blinded	US Canada 118	1000 497 RM 503 UC	12 months	71 (64–77) years 38% female	Any LVEF NYHA II-IV Recent HFH OR elevated natriuretic peptides OMT as tolerated	Candidates for HTx, LVAD or hospice care	CardioMEMS Abbott	Daily PAP data used to guide patient Mx	1º: Composite of all-cause mortality and total HF events (HFH and urgent HF hospital visits)	1º: Not statistically significant. 253 in RM group vs 289 in UC (0.563 vs 0.640 per patient year) (*HR* 0.88, 95% *CI* 0.74–1.05; *p* = 0.16) *Pre-specified pre-covid-19 impact analysis 177 vs 224 (0.533 Vs 0.682 per patient yr) — *HR* 0.81, 95% *CI* 0.55–1.00; *p* = 0.049*
*MEMS-HF *[47•]	2020 Cohort study	Europe 31	234	12 months	68 ± 11 years 22% female	Any LVEF NYHA III HFH in last 12 months	Candidates for HTx, LVAD or hospice care	CardioMEMS Abbott	Weekly PAP data Managed according to pre-defined algorithms	1º: Freedom from device- or system- related complications at 1 year 2º: HFH 12 months post—vs 12 months pre-implant. PAP, KCCQ	1º: Device/system 98.3% (95% *CI* 95.8–100.0) and sensor 99.6% (95% *CI* 97.6–100.0) 2º: Significantly reduced HFH 0.60 vs 1.55 event/patient years post implant vs pre-implant. *HR* 0.38, (95% *CI* 0.31–0.48) *P* < 0.0001* 2º: Significant reduction in PAP of 5.1 ± 7.4 mmHg, KCCQ scores significantly increased
*CardioMEMS PAS *[53]	2020 Cohort study	US 104	1200	12 months	69 ± 12 years 38% female	Any LVEF NYHA III HFH in last 12 months	Candidates for HTx, LVAD or hospice care	CardioMEMS Abbott	Daily PAP data Managed according to pre-defined algorithms	1º: Difference between rates of HFH 12 months post- vs 12 months pre-implant 2º: Freedom from device- or system-related complications and pressure sensor failure at 2 years	1º: Significantly reduced 0.54 vs 1.25 events/patient-years post-implant vs pre-implant, *HR* 0.43 (95% *CI*, 0.39–0.47), *P* < 0.0001* 2º: Device/system 99.6%, pressure 99.9%
*COAST *[48]	2022 Cohort study	UK 14	100	12 months	69 ± 12 years 30% female	Any LVEF NYHA III HFH in last 12 months	Candidates for HTx, LVAD or hospice care	CardioMEMS Abbott	Daily PAP data. Managed according to clinician judgement	1º: Freedom from device related complications and pressure system at 2 years 1º: Rate of HFH in 12 months post- vs 12 months pre-implant	1º: 100% freedom from device related complications and 99% freedom from pressure sensor failure at 2 years 1º: Significantly reduced 0.27 vs 1.52 events/patient-yr post-implant vs pre-implant. 82% risk reduction in HFH (*IRR* 0.18 [95% *CI* 0.12–0.28]; *P* < 0.0001*)

*No*, number; *SD*, standard deviation; *IQR*, interquartile range; *RCT*, randomised controlled trial; *Intl*, International; *RM*, remote monitoring; *UC*, usual care; *LVEF*, left ventricular ejection fraction; *NYHA*, New York Heart Association classification of heart failure; *HFH*, heart failure hospitalisation; *COPD*, chronic obstructive pulmonary disease; *PAH*, pulmonary artery hypertension; *CVE*, cardiovascular event; *Mx*, management; *HF*, heart failure; *CI*, confidence interval; *VTE*, venous thromboembolism; *CIED*, cardiac implantable electronic device; *eGFR*, estimated glomerular filtration rate; *PAP*, pulmonary artery pressure; *HR*; hazard ratio; *OMT*, optimal medical therapy; *HTx*, heart transplant; *LVAD*, left ventricular assist device; *KCCQ*, Kansas City Cardiomyopathy Questionnaire

The pivotal American CHAMPION trial randomised patients with NYHA class III HF across a range of ejection fraction and reported a 30% reduction in HF hospitalisation risk at 6 months [50]. This study, along with other post-marketing randomised and observational studies in the USA and Europe [47•, 48, 53, 54], has suggested robust evidence of benefit when used in specialist centres.

More recently, two European studies have shown the likely added benefit in NYHA class III patients in routine care within healthcare systems outside the USA: MEMS-HF (CardioMEMS European Monitoring Study for Heart Failure) in Germany [47•] and COAST (CardioMEMS HF System Post-Market Study) in the UK [48], with a 62% and 82% reduction in annualised HF hospitalisation rates, respectively. Such data have facilitated positive reimbursement decision making outside the USA, including most recently in England by the National Institute for Health and Care Excellence (NICE) [55]. In Germany, health authorities are awaiting the results of PASSPORT-HF (Pulmonary Artery Sensor System Pressure Monitoring to Improve Heart Failure Outcomes [56]), a randomised study which compares the efficacy of standard HCP-coordinated HF care (“basic care”) plus PAP-guided management with basic care alone, before a final reimbursement decision is made.

Broadening of the indication for remote PAP monitoring using CardioMEMS™ to less symptomatic patients (NYHA class II) was the purpose of GUIDE-HF (Haemodynamic-guided management of heart failure) [52]. Complicated by the COVID-19 pandemic, with huge shifts in patient care patterns during “lockdowns”, the study failed to reach statistical significance overall. A pre-specified pre-COVID analysis (using the majority of data in the study, prior to the first USA COVID-19-related lockdown) suggested a strong evidence of benefit across this broader range of patients (24% reduction in HF events over 12 months, *p* = 0.014). The FDA has just approved the expansion of the use of CardioMEMS to include patients with NYHA class II HF, provided they have experienced a recent HFH or have an elevated plasma natriuretic peptide concentration [57].

The most recent update to the European Society of Cardiology (ESC) HF guideline makes the recommendation for implantable remote monitoring (for CIEDs or haemodynamic monitoring) only for those with the CardioMEMS™ system, and limited to those with NYHA Class III symptoms despite optimal medical therapy [22], while Australian and Canadian guidelines have a weak or no recommendation, respectively [23, 24].

Ongoing studies may expand the choice of location of invasive monitoring device beyond PAP sensors, allowing a more personalised approach related to the individual’s physiology. Implant sites currently under investigation in first-in-human safety trials, include the inferior vena cava (FUTURE-HF) and the left atrium (VECTOR-HF). Placed in the inter-atrial septum, preliminary results from the left atrial pressure sensor show it is likely to be safe, the readings show a strong correlation with invasive PAP measurements and there is a signal of improvement in NYHA class [58].

**Fig. 3:**
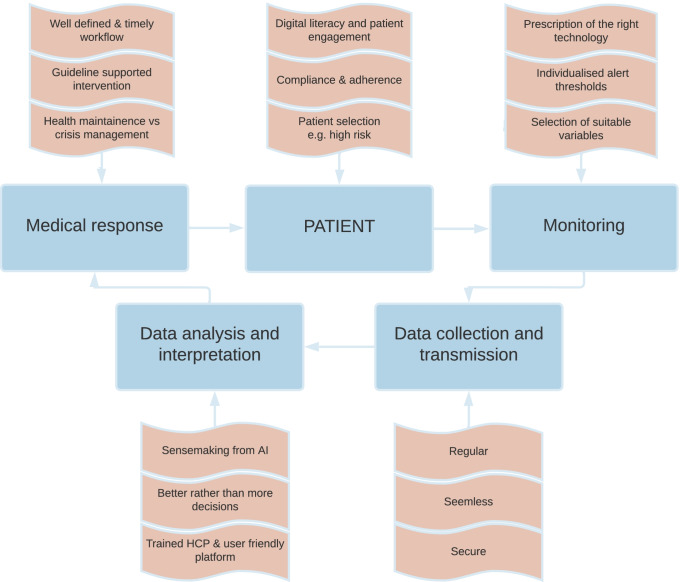
Key elements to consider for successful remote patient management. Modified with permission from Angermann C, 2019 [59]. AI, artificial intelligence; HCP, healthcare professional

A recurring theme in remote monitoring studies is that the ultimate decision maker remains the patient. Patients should be selected with care. They should be at risk of HFH, must “buy in” to the concept of daily collection of data (often requiring their active participation), and must be willing to comply with treatment or adherence recommendations even if asymptomatic. This care cycle will otherwise be broken — and this is particularly likely to happen if patients are contacted by someone they have not met and with whom they do not have a therapeutic relationship [59]. Similarly, HCPs need to be encouraged to act, and to persuade the patient to change therapy (or to be more adherent to therapy) even if they are initially reluctant. Without these actions the potential benefit of early detection of decompensation may be lost — with time taken to collect and review data but without any action being triggered that may reduce risk [59]. HCPs quickly lose interest in reviewing remotely collected data if they cannot observe the benefits for their patients or the system. These and other key elements of RPM are summarised in Fig. 3.

### *Wearables (Table **4**)*

**Table 4:** Trials with wearables

	*Year of publication* *Type of study*	*Location of study* *No. of centres*	*No. of patients* *Intervention group* *Control group*	*Length of follow up* *Mean* ± *SD* *OR Median (IQR)*	*Age* *Mean* ± *SD* *OR Median* *(IQR)* *Sex % female*	*Inclusion criteria*	*Main exclusion criteria*	*Equipment used*	*Intervention*	*Primary (1º) endpoint* *Main secondary (2º) endpoints*	*Effect size*
*SEARCH-AF *[60]	2014 Cross-sectional study	Australia 10 pharmacies	1000		76 ± 7 years 56% female	> 65 years Entering one of the pharmacies	Severe coexisting medical condition preventing participation	AliveCor Kardia single lead ECG (iECG)	If AF, referred to GP	1º: Determine proportion of participants with newly identified AF 2º: Cost-effectiveness analysis of the ICER per QALY gained and CVA avoided for screening vs UC	1º: Newly identified AF was found in 15 participants (1.5%; 95% *CI*, 0.8–2.5%). Of these, 10 participants (1.0%; 95% *CI*, 0.5–1.8%) had no history of AF 2º: If iECG screening was extended into the community the ICER would be €3,142; $USD 4,066) per QALY gained and €15,993; $USD20,695 for prevention of one CVA
*REHEARSE-AF *[61•]	2017 RCT	UK Primary care practices	1001 500 iECG 500 UC	12 months	73 ± 5 years 53% female	> 65 years CHADS-VASc score ≥ 2	Known AF Contra-indication to DOAC CIED	AliveCor Kardia single lead ECG (iECG) and Wi-Fi enabled iPod	Twice weekly 30 s recordings + additional if symptomatic	1º: Time to diagnosis of AF	1º: 19 patients in iECG group were diagnosed with AF vs 5 in UC group (HR 3.9; 95% *CI* = 1.4–10.4; *P* = 0.007) at a cost per AF diagnosis of $10 780 (£8255) 2º: Similar number of CVA/TIA events (6 vs 10, iECG vs UC; *HR* = 0.61; 95% *CI* = 0.22–1.69; *P* = 0.34)
*Apple heart study *[62]	2019 Cohort study	US (Canada) 51 states	420,000	117 (113–186) days	41 ± 13 years 42% female	> 22 years Own an iPhone + iWatch	Previous AF or current use of DOAC/warfarin	None provided	Irregular pulse notification led to telemedicine appointment & ECG patch to confirm AF	1º: AF greater than 30 s on ECG patch monitoring in a participant who received an irregular pulse notification	1º: 0.5% had a positive notification and 34% of these then had AF diagnosed by an ECG patch (95% *CI*, 29 to 39) 1º: PPV for irregular pulse notification of 0.84 (95% *CI*, 0.76 to 0.92) among those who had received an irregular pulse notification
*SCREEN-AF *[63]	2021 RCT	US 48 Primary care practices	856 434 screening group 422 UC	6 months	80 ± 4 years 57% female	≥ 75 years Hypertension	Known AF CIED	Zio-XT patch	2-week continuous ECG patch monitor at baseline & 3 months + UC	1º: AF detected by cECG monitoring or clinically within 6 months 2º: included DOAC use, device adherence, and AF detection by blood pressure monitors	1º: AF detected in 23 of 434 participants (5.3%) in screening group vs 2 of 422 (0.5%) in UC group (*RR*, 11.2; 95% *CI*, 2.7–47.1; *P* = 0.001*; absolute difference, 4.8%; 95% *CI*, 2.6–7.0%; *P* < 0.001*; number needed to screen, 21) 2º: At 6 months, DOAC prescribed for 18 (4.1%) in patch group vs 4 (0.9%) in UC group (*RR*, 4.4; 95% *CI*, 1.5–12.8; *P* = 0.007*; absolute difference, 3.2%; 95% *CI*, 1.1–5.3%; *P* = 0.003*)
*LINK-HF *[64••]	2020 Cohort study	US 4	100	3 months	68 ± 10 years 2% female	Any LVEF NYHA II-IV Current HFH	Visual/cognitive impairment	Reusable sensor, disposable patch and disposable battery Vital Connect	Wear the sensor 24 hr a day, for a minimum of 30 days, and up to 90 days post-discharge	1º: HF readmission after the index discharge from HFH 2º: Time from alert to HFH	1º: The platform was able to detect the risk of HFH with 76.00 to 87.5% sensitivity and 85% specificity 2º: Clinical alerts preceded the hospitalisation by a median time between 6.5 and 8.5 days

*No*, number; *SD*, standard deviation; *IQR*, interquartile range; *RCT*, randomised controlled trial; *AF*; atrial fibrillation; *ICER*, Incremental cost-effectiveness ratio; *QALY*, quality adjusted life-year; *CVA*, cerebrovascular accident; *UC*, usual care; *CI*, confidence interval; *DOAC*, direct oral anti-coagulant; *HR*, hazard ratio; *TIA*, transient ischaemic attack; *PPV*, positive predictive value; *CIED*, cardiac implantable electronic device *UC*, usual care; *LVEF*, left ventricular ejection fraction; *NYHA*, New York Heart Association classification of heart failure; *HFH*, heart failure hospitalisation; *RR*, relative risk; *LVEF*, left ventricular ejection fraction; *NYHA*, New York Heart Association classification of heart failure; *HFH*, heart failure hospitalisation

Wearable medical devices (“wearables”) can provide a variety of data from sensors that typically can be worn on the wrist, clipped to clothing or stuck to the skin [65, 66••]. The most studied are “smart” watches, activity monitors and monitoring patches.

Smartwatches and activity monitors typically combine accelerometers, which track movement, with photoplethysmography (PPG), an optical sensor able to monitor heart rates. PPG traces can also be used to assess cardiac rhythm. The Apple™ Heart study used PPG with an “irregular pulse algorithm” to screen patients for atrial fibrillation (AF); 34% of patients with a positive notification subsequently had AF diagnosed on ECG patch testing [62]. The accuracy of PPG is hindered by a high dropout rate, changes in position, changes in exercise and rapid changes in heart rate [67–69], and that it does not produce an ECG [70]. Despite this, the early results from the Fitbit Heart Study add to the Apple™ Heart study showing the potential utility of wrist-worn wearables in screening of asymptomatic individuals, with likely higher relevance for those at higher risk of AF such as patients with heart failure [60], and those at risk of development of the heart failure syndrome due to AF [71].

ECG sensors, such as Kardia™, have a larger footprint for electrodes allowing recording of up to six ECG leads, improving both diagnostic accuracy and time to diagnosis with beneficial reductions in cost when used for high-risk patients in the community or attending primary care [60, 61•]. This technology can be used with a range of smartphones and NICE has recently recommended this as a validated option in patients with suspected paroxysmal AF [72], allowing patients with HF and palpitation or syncope to benefit from high-quality prolonged rhythm detection.

Wearable patch technology is typically placed on the chest wall and can monitor several additional variables including movement, temperature and respiratory rate for a period of days to weeks. This technology greatly increases the diagnostic yield for AF [63] but also provides data for multiparametric scores similar to those used with remote monitoring of CIEDs in HF.

LINK-HF [64••] (Multisensor Non-invasive Remote Monitoring for Prediction of Heart Failure Exacerbation) studied the VitalConnect™ disposable patch sensor (7-day battery life) with a re-usable sensor electronics module. One module can provide months’ of continuous monitoring. In patients recently discharged after a HFH, the technology was able to identify the risk of further HFH with greater than 76% sensitivity and an 85% specificity in a retrospective analysis — similar to the performance of Medtronic’s Heart Failure Risk Score (HFRS™) and Boston Scientific’s Heartlogic™ algorithm in their development and validation studies in CIEDs [33, 37]. Such patch technology may not just be beneficial in monitoring patients at home but also may allow real-time monitoring in hospital and other care settings, although further validation is required [73].

### *Technologies in Development (Table **5**)*

**Table 5:** Trials with future technology

	*Estimated completion date* *Type of study*	*Country* *No. of centres*	*No. of patients* *Age* *Mean* ± *SD* *OR* *Median (IQR)*	*Length of follow*-*up*	*Inclusion criteria*	*Main exclusion criteria*	*Name of technology* and *company* *Type of technology*	*Intervention*	*Primary (1º) endpoint* *Main secondary (2º) endpoints* *Preliminary data*
*The ReDS-SAFE HF study* *NCT04305717*	Dec 2021 RCT	US 1	240	30 days	Any LVEF Current HFH NT-pro BNP ≥ 400 pg/ml or BNP ≥ 100 pg/ml	height < 155 cm or > 190 cm, BMI < 22 or > 39 kg/m²	ReDS™ Sensible Medical Remote Dielectric Sensor	Daily ReDS measurements with predefined Mx algorithm, discharge when ReDS value ≤ 35%	1º: Composite of unplanned visit for WHF that led to the use of IV diuretics, HFH, or death from any cause at 30 days after discharge Preliminary data SMILE-HF [74]
*Proactive-HF* *NCT04089059*	May 2024 RCT	US 49	970	12 months	Any LVEF NYHA III HFH in last 12 months + NT-proBNP ≥ 1500 pg/mL if HFpEF ≥ 800 pg/mL if HFrEF	CVE in last 3 months	Cordella™ Endotronix Pulmonary artery pressure sensor	Daily PAP guided HF Mx	1º: Mortality and HFH or WHF requiring IV diuretic 1º: Safety: Device/system related complication and pressure sensor failure Preliminary data SIRONA [75]
*ANTEHM-HFrEF *[76] *NCT03425422*	Dec 2024 RCT	US UK 27	800	2 years	LVEF ≤ 35% NYHA II-III HFH in last 12 months NT-proBNP ≥ 800 pg/ml	Systolic BP < 90 mmHg, non-ischaemic HF < 6 months Significant valvular abnormality	Vitaria® System LivaNova Vagal nerve neuromodulator	Chronic stimulation of the right cervical vagus nerve (VNS) Visits for VNS up titration over a period of 3 months	1º: Event-free rate — cardiovascular mortality and HFH Preliminary data ANTHEM-HF [77]
*ALLEVIATE-HF* *NCT04452149*	March 2024 RCT	US 60	700 1:1	7–36 months	Any LVEF NYHA II-III	CIED or severe comorbidity	Reveal LINQ ™ Medtronic Insertable cardiac monitor (ICM)	Managed with integrated device diagnostic-based risk stratification algorithm	1º: Safety of patient Mx pathway 1º: Efficacy of patient Mx pathway — hierarchical composite of Cardiovascular death, HF events, change in KCCQ and 6MWT Preliminary data IDENTIFY-HF
*REVeAL-HF* *NCT03845660*	Dec 2024 RCT Single blinded	US 1	4000	1 year	Any LVEF HFH with IV diuretics within 24 h of admission + NT-proBNP > 500 pg/ml	None	Electronic health record (HER) Alert vs non alert	Provide clinicians with risk of inpatient mortality and 1 year mortality Test clinical impact of providing prognostic information to provider in inpatient setting	1º: All-cause mortality and 30-day risk of HFH 2º: Length of stay, discharge doses of therapies
*HEARTLINE* *NCT04276441*	March 2025 RCT	US	150,000	3 years	> 65 years Owns an iPhone 6 s or later	Limited life expectancy	Apple watch Apple™ Smartwatch	Using Apple watch to investigate if early AF diagnosis reduces the risk of thromboembolic events in the real world	1º: Time from randomisation to clinically confirmed diagnosis of AF 1º: Percentage days covered by DOAC prescription
*MindMics *[78] *NCT05103579*	Nov 2021 Cohort study	US 1	29	30 min	AF as inpatient or outpatient	None	MindMics MindMics Inc. Earbuds with infrasonic haemodynography	Performance of the MindMics device for detecting AF based on inter-beat intervals	1º: Development of algorithm using the MindMics recording system to discriminate AF from sinus rhythm
*C-MIC-II* *NCT04662034*	Feb 2023 Randomised open label	Europe 11	92	6 months	LVEF ≥ 25 and ≤ 35% NYHA II-IV Idiopathic DCM HF diagnosis > 1 yr and < 5 yrs	> 75 years old	C-MIC system Berlin Heals Cardiac microcurrent therapy system	Performance and safety of microcurrent system	1º: Change of the LVEF from baseline Preliminary data C-MIC I [79] first in human study. Rapid and significant signal of efficacy (*P* < 0.005) was present with improvements in LVEF and 6MWT
*Fitbit heart study *[80] *NCT04380415*	March 2021 Open label single arm	US 2	450,000 47 years	7 days	Adults > 22 yrs old	AF CIED	Fitbit® Google Fitness tracker or smartwatch	Validate Fitbit PPG Rhythmdetect software algorithm for providing notifications by identifying rhythms suggestive of AF of atrial flutter	1º: Positive predictive value of the first irregular heart rhythm detection during ECG monitoring: 30 s or more of AF/flutter Of the 4728 irregular heart rhythm detections, 1057 individuals underwent subsequent ECG monitoring. Of the 1057 who underwent ECG patch monitoring, atrial fibrillation was detected in 32.2% (*n* = 340)
*HearO *[81]	Jan 2022 Open label single arm	Israel 1	40 75 ± 12 years	Length of admission	Any LVEF Any NYHA Current HFH	MI, eGFR < 25 ml/min, ESRF, mechanical valve	HearO™ Cordio Medical Voice capturing application	Patients admitted with acute decompensated HF (wet) record 5sentences into a smartphone and then again at discharge (dry). These were analysed with 5 distinct speech measures (SM)	1º: Difference and correlation of fluid status identifying speech measures with pre-dialysis and post-dialysis fluid status Interpatient comparisons of collected recordings identified significant differences in all 5 tested SMs of wet (admission) vs dry (discharge) recordings (*P* < 0.0001*)
*BMAD-TX* *NCT03476187*	March 2022 Open label single arm	Austria Germany US	500	6 months	Any LVEF Any NYHA Current or recent (10 days) HFH	S-ICD, < 1 year life expectancy, ESRF	µCor patch ZOLL® Radiofrequency technology	Weekly μCor data and phone call Clinic visit day 30, 60 and 90 days	1º: Correlation of µCor measured thoracic fluid index to HF related clinical events 2º: Correlation of other measured parameters to related clinical events
*VisONE *[82]	Oct 2020 First in human safety study	Intl	15 60 (56–67) years	12 months	LVEF ≤ 35% Any NYHA Sinus rhythm with narrow QRS	Severe COPD	VisONE® VisCardia Synchronised diaphragmatic stimulator (SDS)	Laparoscopic implantation of VisONE SDS system Modulate pressure in intra-thoracic cavity	1º: Procedural success and freedom from therapy related complications Between discharge with SDS off and SDS on at 3, 6 and 12 months, improvements seen in exercise capacity, SF-36 and LVEF with larger effects when diaphragmatic synchronisation was > 80%
*FUTURE-HF* *NCT04203576*	April 2022 First in human safety study	Czech Republic	10	3 months	Any LVEF NYHA III HFH or IV diuretics or urgent outpatient visit in last 12 months	Significant co-morbidity eGFR < 30 ml/min	FIRE1 system Foundary Innovation & Research 1 IVC pressure sensor	FIRE1 sensor implant	1º: Procedural success and freedom from FIRE1 sensor complications 1º: Technical Endpoint — signal acquisition from the FIRE1 sensor Changes in IVC represent a sensitive measure of intravascular volume and tone [83]
*Vector-HF *[58] *NCT03775161*	Dec 2024 First in human safety study	Germany Italy UK	45	3 months	Any LVEF NYHA III HFH in last 12 months Maximal OMT 3 months	End-stage HF, hypotension > 85 years	V-LAP™ Vectorious medical technologies Ltd Left-atrial pressure sensor	V-LAP™ implant via RHC Daily left atrial pressure measurements	1º: Ability to successfully deliver (to the interatrial septum) and deploy the V-LAP™ implant. Safety Endpoint: Device and/or system related Major Adverse Cardiac and Neurological Events 24 patients: 100% procedural success, no device-related complication. LAP correlated with wedge pressure (*R* = 0.86). NYHA functional class was better than at baseline at 6 months (2.6 ± 0.6 vs 3.0 ± 0.0; *P* = 0.007)
*Nanosense* *NCT03719079*	Dec 2021 Cohort study	US 10	500	90 days	Any LVEF NYHA II-IV Current or recent HFH	Severe aortic stenosis or angina Clinically unstable	SimpleSense Nanowear Wearable congestive HF Mx system	Wear device 12 h daily including 2 h prior to sleep and 2 h after awakening	1º: Develop and validate a multi-parameter algorithm for the detection of HF prior to HFH

No, number; SD, standard deviation; IQR, interquartile range; RCT, randomised controlled trial; LVEF, left ventricular ejection fraction; NYHA, New York Heart Association classification of heart failure; HFH, heart failure hospitalisation; BMI, body mass index; WHF, worsening heart failure; CVE, cardiovascular event; PAH, pulmonary artery hypertension; HF, heart failure; Mx, management; CIED, cardiac implantable electronic device; KCCQ, Kansas City Cardiomyopathy Questionnaire; 6MWT, six-minute walk test; AF, atrial fibrillation; DOAC, direct oral anti-coagulant; DCM, dilated cardiomyopathy; PPG, photoplethysmography; MI, myocardial infarction; eGFR, estimated glomerular filtration rate; ESRF, end-stage renal failure; S-ICD, subcutaneous implantable cardiac defibrillator; COPD, chronic obstructive pulmonary disease; SF-36, short form quality of life questionnaire; RHC, right heart catheterisation

Examples of novel technologies under investigation to improve RPM in HF include: ReDS (remote dielectric sensor from Sensible Medical), SimpleSense™ (Nanowear), µCor™ (Zoll®) and HearO™ (Cordio Medical).

ReDS uses an electromagnetic based technology developed by the military to provide an instantaneous estimation of lung fluid content. Using ReDS to guide management in recently discharged patients with HF demonstrated a halving of the rate of HFH over 6 months in a non-randomised study [74]. The ReDS-SAFE HF trial is currently randomising up to 240 patients admitted for HF at one US centre, to ReDS-guided discharge or usual care, with a primary composite endpoint of unplanned visit for HF or all-cause mortality (NCT04305717).

SimpleSENSE™ is an FDA-cleared wearable undergarment that monitors several vital signs such as heart rate, heart sounds, respiratory rate, lung volume and physical activity and is connected to a machine-learning platform. Results are awaited from a cohort study developing and validating a multi-parametric algorithm to detect HF decompensation.

µCor is an ECG patch and HF management system that uses radiofrequency technology to measure multiple parameters including thoracic fluid index. A multicentre trial aims to correlate this to the risk of HF events (NCT03476187).

Voice detection algorithms allow the HearO™ mobile phone application to assess fluid status and highlight the difference between pre- and post-dialysis states [84], and through fluid optimisation during a HFH [81].

## Factors Affecting the Development and Use of Digital Tools in HF Care

**Fig. 4:**
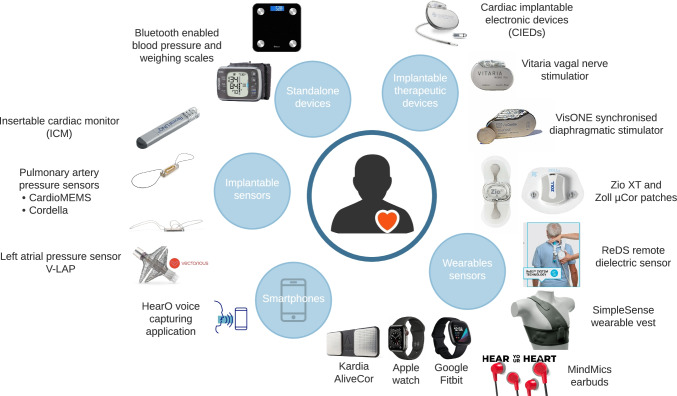
The range of digital technologies that patients with heart failure potentially have available to them

There are an increasing range of digital technologies available to support remote patient management in HF (and other diseases) (Fig. 4) but several factors are pivotal to their optimal development, maintenance, and longer term use. We have expanded on these issues in our commentary in this edition of the journal, entitled *The Digital Future of Heart Failure Care*. They include issues around the evaluation and regulation of technology, co-design and co-implementation, data security and access, machine learning in support of decision-making and litigation risk. Key to success is a better understanding of the support needs and capabilities of patients living with HF (and their healthcare advisors) including their digital and health literacy and degree of activation.

## Right Technology, Right patient, Right time: a Vision of the Future

The traditional model of HF care with periodic clinic review, using only data collected at that time by the healthcare team using “their” technologies, is likely to be replaced (or at least supplemented) by a more patient-centric approach.

Collecting data remotely provides “real” life data — which represent more than 99% of the time when patients are in their own environment — to be used in shared decision-making. Shared decision-making requires a good working relationship between patient and HCP, as well as a patient who is adequately informed, educated and (therefore) motivated to make changes based on remote monitoring data. Access to HCPs and systems will still be needed and must be available flexibly and at potentially short notice, but much of the routine processes of care can be supported by technology and RPM, freeing up time and bandwidth for members of the healthcare team to deal with the more complex, or nuanced, situations where human-to-human interaction adds more value.

In practice, digital remote technologies will support the key activities within the care pathway — ensuring rapid and accurate diagnosis, risk stratification and prognostication, therapeutic decision-making and tailored support including education and care. Active shared decision-making between clinician and patient will decide which technology can best be used to achieve the shared goals, whether it is initial education and support to self-care, identification when another therapy may be beneficial, or helping the patient maintain stability with optimised quality of life at home, without the need to engage with the “official” healthcare system by attending clinic or being hospitalised.

Technology should provide closed-loop interactions with patients, offering advice on fluid intake, diuretic regime, exercise, necessity for blood tests and when to contact an HCP. When patients require input from an HCP they deserve a tailored and responsive interaction based on multiple relevant data points and sources, with decision and sensemaking supported by digital tools (and likely artificial intelligence). Clinical deterioration will be predicted and identified early using remote monitoring and stabilisation facilitated by appropriate escalation of therapy. Admission to hospital will, of course, remain necessary for some patients, but early discharge will be supported with virtual wards (“hospital-at-home”) with monitoring pathways and rehabilitation facilitated remotely.

We highlight the differences between the traditional model of care and this more modern, digitally enabled approach to HF care in Fig. 1.

## Conclusion

Digital technologies are already deeply embedded within all aspects of society, including health and healthcare. HF care already relies on many of them, but the clinical pathways and treatment algorithms to successfully integrate digital technologies and decision support into the healthcare system are in their infancy.

Ultimately, digital technologies will become part of the “new normal”, being selected for use when they make sense and help patients and their HCPs achieve shared goals. Due consideration must be paid to ensuring the evidence base is robust, that data flows and human factors are considered, and that the digital technologies are co-designed and implemented to ensure a better outcome and experience of care. Data should not be collected for its own sake — it must support better decision-making and more efficient care.
